# Implementing alcohol use disorder pharmacotherapy in primary care settings: a qualitative analysis of provider-identified barriers and impact on implementation outcomes

**DOI:** 10.1186/s13722-019-0151-7

**Published:** 2019-07-10

**Authors:** Hildi J. Hagedorn, Jennifer P. Wisdom, Heather Gerould, Erika Pinsker, Randall Brown, Michael Dawes, Eric Dieperink, Donald Hugh Myrick, Elizabeth M. Oliva, Todd H. Wagner, Alex H. S. Harris

**Affiliations:** 1 Veterans Affairs Health Services Research and Development Center for Care Delivery and Outcomes Research, Minneapolis Veterans Affairs Health Care System, Minneapolis, MN 55417 USA; 2 Department of Psychiatry, University of Minnesota School of Medicine, Minneapolis, MN 55455 USA; 3 Jennifer Wisdom Consulting, New York, New York, NY 10010 USA; 4 William S. Middleton Memorial Veterans Hospital, Madison, WI 53705 USA; 5 Department of Family Medicine, University of Wisconsin School of Medicine and Public Health, Madison, WI 53715 USA; 6 Substance Abuse Treatment Program, South Texas Veterans Affairs Health Care System, San Antonio, TX 78229 USA; 7 Department of Psychiatry, University of Texas Health Science Center at San Antonio, San Antonio, TX 78229 USA; 8 Center for Drug and Alcohol Problems, Ralph H. Johnson Veterans Affairs Medical Center, Charleston, SC 29401 USA; 9 Department of Psychiatry and Behavioral Services, Medical University of South Carolina, Charleston, SC 29425 USA; 10 Veterans Affairs Health Services Research and Development Center for Innovation to Implementation, Palo Alto Veterans Affairs Health Care System, Menlo Park, CA 94025 USA; 11 Health Economics Resource Center, Palo Alto Veterans Affairs Health Care System, Menlo Park, CA 94025 USA

**Keywords:** Alcohol use disorder pharmacotherapy, Primary care mental health integration, Implementation, Implementation barriers

## Abstract

**Background:**

Despite the high prevalence of alcohol use disorders (AUDs), in 2016, only 7.8% of individuals meeting diagnostic criteria received any type of AUD treatment. Developing options for treatment within primary care settings is imperative to increase treatment access. As part of a trial to implement AUD pharmacotherapy in primary care settings, this qualitative study analyzed pre-implementation provider interviews using the Consolidated Framework for Implementation Research (CFIR) to identify implementation barriers.

**Methods:**

Three large Veterans Health Administration facilities participated in the implementation intervention. Local providers were trained to serve as implementation/clinical champions and received external facilitation from the project team. Primary care providers received a dashboard of patients with AUD for case identification, educational materials, and access to consultation from clinical champions. Veterans with AUD diagnoses received educational information in the mail. Prior to the start of implementation activities, 24 primary care providers (5–10 per site) participated in semi-structured interviews. Transcripts were analyzed using common coding techniques for qualitative data using the CFIR codebook Innovation/Intervention Characteristics, Outer Setting, Inner Setting, and Characteristics of Individuals domains. Number and type of barriers identified were compared to quantitative changes in AUD pharmacotherapy prescribing rates.

**Results:**

Four major barriers emerged across all three sites: complexity of providing AUD pharmacotherapy in primary care, the limited compatibility of AUD treatment with existing primary care processes, providers’ limited knowledge and negative beliefs about AUD pharmacotherapy and providers’ negative attitudes toward patients with AUD. Site specific barriers included lack of relative advantage of providing AUD pharmacotherapy in primary care over current practice, complaints about the design quality and packaging of implementation intervention materials, limited priority of addressing AUD in primary care and limited available resources to implement AUD pharmacotherapy in primary care.

**Conclusions:**

CFIR constructs were useful for identifying pre-implementation barriers that informed refinements to the implementation intervention. The number and type of pre-implementation barriers identified did not demonstrate a clear relationship to the degree to which sites were able to improve AUD pharmacotherapy prescribing rate. Site-level implementation process factors such as leadership support and provider turn-over likely also interacted with pre-implementation barriers to drive implementation outcomes.

**Electronic supplementary material:**

The online version of this article (10.1186/s13722-019-0151-7) contains supplementary material, which is available to authorized users.

## Background

In 2016, 15.1 million adults in the US (5.6%) met diagnostic criteria for an alcohol use disorder (AUD) and 6% of the population engaged in heavy drinking (5 or more drinks for men and 4 or more drinks for women on 5 or more days out of the past 30 days) [1]. AUDs and heavy drinking are associated with car crashes, domestic violence, neurocognitive impairments, poor medication adherence, psychiatric comorbidity, and increased morbidity and mortality [2–8]. The total societal costs of AUDs and heavy drinking, including health care costs, law enforcement costs, other direct costs (e.g., material losses due to accidents), and productivity losses were over $131 billion dollars in 2016, representing 1% of the total US Gross Domestic Product [9]. Despite the high prevalence and costs associated with AUDs, treatment rates in the general population remain astonishingly low. In 2016, only 7.8% of individuals meeting diagnostic criteria for AUD received any type of AUD treatment [1]. Improving access to evidence-based treatments for AUD has the potential to reduce suffering and realize savings in health care costs.

The under-treatment of and costs associated with AUD are also major problems within the Veterans Health Administration (VHA). The VHA provides care to over 300,000 Veterans with AUD diagnoses annually, yet only approximately one-third of these patients receive treatment in one of VHA’s 220 specialty addiction programs [10, 11]. While it is possible that some of these patients receive treatment for their AUD outside of the VHA, given the low treatment rates for AUD within the general population, it is unlikely that non-VHA treatment is substantially addressing this issue. Yu and colleagues found that the added medical cost associated with an AUD diagnosis was $3124 (1999 dollars) per patient per year [12].

Clearly, different care delivery models are needed to offer and increase accessibility of treatment for individuals with AUD. Although the need is great and interest in integration of AUD treatment into primary care settings is high, few models for implementation of AUD treatment into primary care have been tested [13–17]. Several of these models have incorporated pharmacotherapy for AUD which is recognized as an important treatment option but continues to be rarely used in clinical care.

Randomized controlled trials and meta-analyses support the efficacy of pharmacological treatment with naltrexone or acamprosate to improve drinking outcomes including length of time to relapse, number of drinking days, and number of drinks per drinking day [18–21]. Use of naltrexone or acamprosate for patients with AUD is supported by National Quality Forum’s National Voluntary Consensus Standards for the Treatment of Substance Use Conditions published in 2007 and the Veterans Administration-Department of Defense Clinical Practice Guidelines for Management of Substance Use Disorders updated in 2015 [22, 23].

Despite the evidence supporting the use of naltrexone and acamprosate for treating AUD, these medications are underutilized in the United States [24], and this underutilization is also seen within the VHA. Among VHA patients with a documented AUD diagnosis, only 8.5% receive any type of approved AUD pharmacotherapy. There is also extreme variability in prescribing rates across the country at the facility level. The proportion of Veterans who received AUD specialty care who also received pharmacotherapy for AUD ranged from 0 to 20% by facility; Veterans with no contact with AUD specialty care have even lower prescribing rates [25]. Extremely low prescribing rates and significant variation across facilities suggests that significant gaps exist in access to and utilization of these medications and that more could be done to increase prescribing rates.

Currently in VHA, virtually all patients are screened for risky alcohol use on an annual basis. Usually, the screener questions are asked by the nurse prior to the provider visit. Providers are alerted to patients who screen positive and are expected to discuss the screen with the patient and, if deemed appropriate, refer the patient for AUD treatment. However, as detailed above, rates of specialty care treatment attendance and rates of pharmacotherapy prescriptions continue to be stubbornly low. The Alcohol use Disorder Pharmacotherapy and Treatment in Primary Care (ADaPT-PC) trial sought to increase implementation of AUD pharmacotherapy in primary care clinics in three large VHA facilities using a multi-faceted implementation intervention targeting multiple stakeholder groups. Details of the methods for the larger project are available in the published protocol [26]. Local substance use disorder specialists and primary care mental health integration providers were trained to serve as local champions and consultants for primary care providers. Primary care providers received access to a website with educational materials regarding management of AUD in primary care settings, contact information for local and national AUD treatment consultants, and a personalized dashboard of their patients with a documented AUD diagnosis in their charts. Primary care providers also received email alerts if a patient from their dashboard had a clinic visit scheduled within the next week. Finally, patients with AUD diagnoses received direct mailing of a brochure describing pharmacotherapy options for AUD treatment.

The original design of the implementation intervention was based on the Theory of Planned Behavior as well as prior research documenting barriers to implementation of AUD treatments in primary care settings [27, 28]. Following previous recommendations for enhancing implementation trials focused on substance use disorders [29], we incorporated an extensive formative evaluation into this project with the intent of contributing to knowledge regarding the determinants of successful implementation. The formative evaluation was guided by the Consolidated Framework for Implementation Research (CFIR), an overarching typology to guide implementation development and verification about what works, where and why across multiple contexts [30]. The CFIR integrates implementation theories and provides consistent definitions and terminology for 39 constructs in five domains: Innovation/Intervention Characteristics, Outer Setting, Inner Setting, Characteristics of Individuals, and Process. The initial step of the formative evaluation was completion of a developmental evaluation which consisted of interviews with primary care providers, patients with AUD, and the identified local project champions to revise and refine the implementation intervention.

While patient knowledge and willingness to discuss AUD treatment with their primary care provider may definitely impact implementation and identified champions may have opinions about how to best modify primary care provider behavior, ultimately it is the primary care provider who must decide if they are willing to discuss AUD, make treatment recommendations and prescribe pharmacotherapy. Therefore, understanding their perceptions of the major barriers to implementation is vital to addressing the gap in AUD treatment. This paper presents the results of qualitative interviews conducted with primary care providers at the three participating sites during the developmental evaluation phase prior to the start of the implementation intervention.

The first aim of this paper is to illustrate how the pre-implementation assessment of barriers informed refinements to the implementation intervention. The second aim is to assess for potential variance in pre-implementation barriers across facilities given the inconsistent effect of the implementation intervention across sites. The results of the quantitative interrupted time-series analysis are detailed elsewhere [31]. Briefly, the results of this analysis indicated a significant increase in AUD pharmacotherapy prescribing at the intervention facilities overall, but not in comparison to secular trends in non-intervention sites. However, results varied by site with one site having a significant increase in prescribing, one site demonstrating a non-significant trend toward an increase in prescribing, and one site demonstrating no change in prescribing. By assessing differences in pre-implementation barriers by impact of the implementation intervention, our goal was to contribute to efforts to identify CFIR constructs that may be more promising candidates for predicting implementation success in future studies.

## Methods

### Participants

Three large, geographically diverse VHA facilities were recruited to participate in the implementation intervention based on the availability of local substance use disorder specialty care providers and primary care mental health integration providers interested in serving as local champions for the project. Every VHA facility is required to have providers who are mental health specialists, which can include psychiatrists, psychologists, and/or social workers, who are physically located within Primary Care clinics and should be available for same-day warm hand-offs. Facilities with identified providers to fill the champions roles were also required to secure approval from the chief of primary care. Table 1 indicates the geographic region and complexity of each facility as well as the credentials and experience of the identified champions. Prior to the start of the implementation intervention, primary care providers were recruited for interviews via email solicitations. Recruitment emails were sent to all primary care providers with prescribing privileges at one of the three participating facilities. Initial emails were followed by reminder emails 1 and 2 weeks following the initial email. There were no exclusion criteria. Interviews were conducted with all providers who responded to the email solicitation with a goal of conducting up to 10 interviews per site. Actual enrollment was 24 providers with 10, 5 and 9 interviews conducted at each site respectively. Table 1 also provides the number of primary care providers at each site, the number of interviews completed and the percentage of total primary care providers interviewed. The percentage of primary care providers interviewed across sites ranged from 10 to 16%. This study received approval from the VHA Central Institutional Review Board. All interview participants provided written informed consent.

**Table 1:** Facility characteristics, champion characteristics and quantitative implementation outcome measures

	Site 1	Site 2	Site 3
*Facility characteristics*			
Geographic region	South	Midwest	Midwest
Complexity*	1A	1B	1A
Number of primary care providers	63	47	93
Number of primary care provider interviews completed	10	5	9
Percent of primary care providers interviewed	16%	11%	10%
*Substance use disorders champions*			
Training	Addiction psychiatrist, board certified in addiction psychiatry and addiction medicine	Primary care physician, board certified in addiction medicine	Addiction psychiatrist, board certified in addiction psychiatry
Years of experience	20	15	30 +
Supervisory role	Addiction medicine fellowship director	Addiction medicine fellowship director	Addiction medicine fellowship director
*Primary care mental health integration champions*			
Training	Clinical pharmacy specialist	Clinical social worker	Clinical psychologist
Years of experience	3	20 +	22
Supervisory role	None	Co-director, primary care mental health integration	Director, primary care mental health integration
Total hours of champion time documented	380	103	82
Pre-implementation prescribing rate	3.6%	4.3%	3.4%
Post-implementation prescribing rate	6.3%	4.6%	5.5%
% Difference	2.7% (ns)	0.3% (ns)	2.1% (p < 0.01)
% Relative increase	75%	7%	62%

*Complexity rating for VHA hospitals employs several variables, including the total number of patients served by the facility, the number and types of intensive care units in the facility, the number of resident programs and the total number of resident slots available, the total amount of research dollars managed by a facility, and the number and breath of physician specialists employed by the facility. 1 = High Complexity (A representing highest level in this category followed by B and C); 2 = Medium Complexity; 3 = Low Complexity

### Procedures

Interviews were conducted by the local project coordinator at each site in the primary care providers’ office space and lasted approximately 60 min. Interviewers all completed an in-person 2 days training on semi-structured interviewing techniques conducted by the research team qualitative expert (JPW). The semi-structured interview guides asked the provider to describe their current practice for screening and treating AUD, gathered feedback on the proposed implementation intervention and provider and patient educational materials, and identified local barriers and facilitators to implementation guided by CFIR constructs ([30]; see Additional file 1 for interview guide). The interviews focused on implementation of medication therapy for AUD in general and did not explore how barriers might differ depending on medication type (naltrexone vs. acamprosate) or formulation (e.g., oral vs. injectable naltrexone).

Interviews were audio recorded and transcribed to create written documents for qualitative coding.

### Data analysis

All transcripts were reviewed by the qualitative team (HH, JPW, HG) to identify common barriers across sites; these barriers were used to inform refinements to the implementation plan. After the implementation intervention concluded, a more in-depth qualitative analysis was conducted to retrospectively identify pre-implementation site-level differences that may have informed the inconsistent effect of the implementation intervention.

All transcripts were entered into a qualitative data analysis program (NVivo) that enables researchers to mark blocks of text with thematic codes and explore relationships among and between codes and groups. Transcripts were analyzed using common coding techniques for qualitative data [32]. Transcripts were coded using the CFIR codebook Innovation/Intervention Characteristics, Outer Setting, Inner Setting, and Characteristics of Individuals domains [33, 34]. CFIR Process domain constructs were not coded as the interviews took place prior to the start of the implementation intervention. The coding strategy allowed for coding a single chunk of text to multiple CFIR constructs if deemed appropriate. The coding strategy also allowed for the addition of inductive emergent codes that identified important themes not represented by the CFIR constructs. The qualitative team added an additional emergent code of “Attitudes Toward Patients” which was used to code the frequent general statements by providers about the behavior or perceived character of patients with AUD diagnoses (see Additional file 2 for codebook.)

Two members of the research team (HH, HG), blinded to the facility from which an interview was collected, coded each transcript separately and then collectively, along with the research team qualitative expert (JPW), came to consensus on coding decisions. Inconsistencies were resolved through discussion and mutual agreement. The original codebook was modified to add exemplar text segments for individual CFIR constructs as the qualitative coding team came to agreement on text segments that would or would not receive a particular CFIR construct code.

Once agreement was reached on coding for all transcripts, code reports were created for each CFIR construct for each facility. Because double coding was allowed, a specific chunk of text could appear on more than one coding report. Only code reports that contained text segments from a minimum of three respondents from that facility were analysed. This protected against idiosyncratic responses and ensured that constructs were addressed by multiple respondents.

Qualitative analysts (HH, EP), remaining blind to facility, then reviewed each code report and rated each CFIR construct for each facility per CFIR recommended guidelines [33, 34] (see Table 2 for scale definitions). The scale is used to assess the valence of the comments within the construct and their influence on implementation. Investigators assigned values ranging from − 2 to + 2, with the addition of an asterisk indicating at least one coded passage that did not conform to the overall rating, e.g., overall positive comments with one negative comment. For code reports where the analysts did not agree on the initial score, consensus meetings were held to resolve disagreement. The consensus process rather than a process of calculating reliability statistics is typically used for CFIR coding given the complexity of the coding scheme [34]. Code reports were then unblinded to create facility-level ratings of CFIR constructs with the goal of identifying differences in the number or type of barriers across facilities that may assist in interpreting the differing effects of the implementation intervention on change in AUD pharmacotherapy prescribing rate during the implementation period.

**Table 2:** Definitions for the CFIR construct rating scale

− 2	Comments are negative AND there is a potential negative impact on implementation
− 1	Comments are negative, but an impact on implementation is unclear or minimal
0	Comments are neutral and construct has no bearing on implementation
+ 1	Comments are positive, but an impact on implementation is unclear or minimal
+ 2	Comments are positive AND there is a potential positive impact on implementation

*Denotes mixed response. An overall rating is agreed on with recognition that at least one respondent’s comments did not match the overall rating

## Results

### Aim 1: Identify barriers common across facilities to inform refinement of the implementation intervention

Table 3 presents CFIR construct rating by facility. Three CFIR constructs received negative ratings for all three facilities, indicating predominantly negatively toned comments with a potential negative impact on implementation: (1) Complexity (Innovation/Intervention Characteristics); (2) Compatibility (Inner Setting); and (3) Knowledge and Beliefs about the Innovation/Intervention (Characteristics of Individuals). The additional emergent code of Attitudes Toward Patients was also negatively rated for all three facilities.

**Table 3:** CFIR construct ratings by site

Site number: prescribing rate change [25]	Site 1: NS trend toward increase	Site 2: No change	Site 3: Significant increase
*CFIR construct*			
Innovation/intervention characteristics domain			
Evidence strength and quality	0	+ 1	+ 1
*Relative Advantage*	+ 1*	− 2*	− 1*
Adaptability	+ 2	–	− 1*
Trialability	+ 2*	+ 2	+ 1*
*Complexity*	− *2*	− *1*	− *1*
*Design quality and packaging*	+ 1*	− 1*	− 1*
Outer setting domain			
Peer pressure	0	0	0
External policy and incentives	0	+ 1	0
Inner setting domain			
Network and communications	+ 1*	+ 1	+ 1*
Culture	− 2*	–	− 1
Implementation climate			
*Compatibility*	− *2*	− *2*	− *2*
*Relative priority*	+ 1*	− *1*	− 1*
Organizational incentives and rewards	0	0	+ 1
Readiness for implementation			
*Available resources*	+ 1*	− *1*	− 1*
Access to knowledge and information	− 1*	+ 1	− 2
Characteristics of individuals domain			
*Knowledge and beliefs about the innovation/intervention*	− 1*	− *1*	− 1*
*Self*-*efficacy*	+ 1*	− 2*	+ 1*
Other personal attributes			
*Attitude toward patients*	− *1*	− *2*	− *2*

The following CFIR constructs had fewer than thee respondents with statements coded to that construct at all three facilities and therefore were not analyzed: Innovation/Intervention Source, Cost, Patient Needs and Resources, Cosmopolitanism, Structural Characteristics, Tension for Change, Goals and Feedback, Learning Climate, Leadership Engagement, Individual Stage of Change, Individual Identification with the Organization

*NS* nonsignificant

*Indicates mixed rating; – indicates < than three respondents at that facility had statements coded to that construct; *Italicized constructs* represent those discussed in the results section

Complexity is defined as “the perceived difficulty of the innovation, reflected by duration, scope, radicalness, disruptiveness, centrality, intricacy and number of steps required to implement” [30]. While the research team had originally conceived of prescribing pharmacotherapy for AUD in primary care as a relatively simple practice change, the providers interviewed perceived a number of steps required to perform this task. Because all primary care providers in VHA are expected to review annual risky alcohol use screening, discuss positive screens with their patients, and refer to specialty AUD care as indicated, it was assumed that primary care providers already possessed sufficient knowledge to make AUD diagnoses. However, interviewees indicated that they were not familiar with the diagnostic criteria. Therefore, they would first have to learn the criteria and then familiarize themselves with how to assess for the criteria during a patient interview. Such assessment would involve asking a series of questions they were unfamiliar with, and in some cases, uncomfortable asking. Then they would also have to educate themselves about medications with which they had little familiarity. Interestingly, none of the interviewees mentioned the potential complexity associated with the fact that positive AUD screen criteria differ for males versus females. This may be because the computerized screener determines whether the screen is positive or negative, therefore the providers may not have even been aware that the criteria differ by gender. They also expressed concerns about how to identify patients at high risk for severe alcohol withdrawal, the potential need for medically supervised withdrawal, and the consequences to the patient if they did not make that identification accurately. A provider’s statement exemplifies these common perceptions:

We’re not considering it as a separate thing from anything else that’s going on with the patient so we have to be really careful in what, who, and why and what we can start on a patient. We need to be very aware of what contraindications there may be, what side effects we might be seeing, how soon they need to be coming in, compliance and everything with these meds. So it is actually tricky. I don’t think we’re ready, that’s what I would say. At a primary care level, I would say. (Primary care provider, Site #1)

Compatibility is defined as “the degree of tangible fit between meaning and values attached to the innovation by involved individuals, how those align with individuals’ own norms, values, and perceived risks and needs, and how the innovation fits with existing workflows and systems” [30]. Most text segments coded to compatibility had to do with lack of fit of prescribing AUD pharmacotherapy with the existing workflows and systems in primary care. Primary care providers reported that, due to their large panel sizes, they generally see each patient for a 20-min appointment every 6 months. They gave priority to the patient’s needs, followed by system-dictated reminders. Providers felt there was inadequate time to evaluate patients’ alcohol use or thoroughly discuss treatment options. They were also concerned that their schedule would not allow for follow-up appointments within a reasonable time frame to assess for the patient’s response to the new medication. These concerns are summarized by the following provider statement:

I would not use it due to time constraints. I can’t think that I ever in my last 6 years met and discussed exclusively alcohol use as the reason for the visit. It comes up embedded in annual visits in which you have lot of other things. (Primary care provider, Site #2)

Knowledge and Beliefs about the Innovation/Intervention is defined as “individuals’ attitudes toward and value placed on the innovation, as well as familiarity with facts, truths, and principles related to the innovation” [30]. This construct does not refer to providers’ evaluation of the evidence base for an intervention, which would fall under the construct of Evidence Strength and Quality. In general, providers at all three facilities agreed that medication treatment for AUD was an evidence-based practice. Instead, this construct refers to both their level of knowledge about the intervention and their attitudes toward it. Level of knowledge of AUD pharmacotherapy was almost universally described as low. With few exceptions, providers reported that they had no training during medical school and no continuing education related to AUD pharmacotherapy. Attitudes toward treating AUD with medications, despite endorsing the medications as evidence-based, were frequently negative. Providers expressed beliefs that treating substance use disorders with medications would not address the underlying causes of the addictive behavior and that behavioral treatments or self-help approaches were the appropriate paths to sustained recovery. For example, this provider suggests that those with alcohol use problems need to deal with the “underlying” problems rather than being prescribed medication for symptom abatement:

If you think [addiction is] maybe partly chemical but maybe also just kind of a maladaptive way that people kind of deal with things in their life that aren’t going well, then giving them a pill seems like maybe not the best route to go or incomplete treatment. So my worry would be so I give someone a pill, so they cut down a little bit but they haven’t really maybe dealt with the underlying issue and if they haven’t developed different coping skills or different ways to deal with that, are they just going to go right back to drinking in the future? Maybe the pill was a little helpful or helpful for a while but not really. (Primary care provider, Site #3)

The final barrier common across all sites was Attitudes Toward Patients, which the qualitative analysts defined as “generalized statements about the behavior or perceived character of the population of individuals with AUD”. Providers frequently made generalized statements about this group of patients as uninterested in getting help, unable or unwilling to follow through with treatment, highly complicated cases with multiple physical and mental health comorbidities, liars and “difficult” patients. One provider, for example, indicates strong negative beliefs about patients’ honesty:

It becomes a sort of fool’s errand to continue to talk about [alcohol use disorder. The patients] are telling you, ‘Oh, I have two drinks a night,’ and you know that it is quite a bit more than that but you can’t really fully call them a liar. (Primary care provider, Site #3)

Several aspects of the implementation intervention were designed to address these four commonly expressed barriers (see Table 4). To address concerns about the intervention complexity, while educational materials had already been developed, much more condensed and simplified versions were created to allow quick access to key information (while still allowing providers to access more detailed materials as desired). The level of concern over managing withdrawal symptoms was greater than expected so simple, quick information on identifying withdrawal risk signs was developed along with recommendations for when to refer to specialty substance use disorder care for management. To address concerns about the compatibility of the intervention with the current processes in primary care settings, the most significant refinement to the intervention was adding in much more extensive interaction with primary care mental health integration services to identify appropriate site-specific processes to allow for “warm handoffs” to assist with AUD diagnosis and with providing more regular follow-up than primary care providers’ schedules allowed. To address gaps in knowledge, providing multiple venues for educational opportunities was always part of the planned implementation intervention. However, the identification of negative beliefs toward pharmacotherapy as a legitimate treatment for AUD led to reframing the educational presentations. Pharmacotherapy was presented as one of many treatment options that a patient might choose from, consistent with the VHA emphasis on patient-centered care and patient choice, with acknowledgement that medications were likely to be most effective when combined with psychosocial interventions. However, medications were framed as a potential “foot-in-the-door” to treatment for patients who were not interested in or turned off by psychosocial treatment options. Finally, to address negative attitudes toward patients with AUD in general, education was provided on the spectrum of AUD from mild to severe and referral to specialty substance use disorder care for the most severe and complex cases was encouraged. The goal was to reduce primary care providers’ initial reluctance to begin treatment conversations about AUD by giving them “permission” to then refer the most complex cases.

**Table 4:** Implementation strategies designed to address commonly identified CFIR barriers

CFIR construct and barrier summary	Implementation strategies to address identified barriers
Intervention characteristics: complexity	
Complexity of steps to diagnose AUD and select appropriate AUD medication prior to prescribing	One page “cheat sheet” of AUD diagnostic criteria One page “cheat sheet” of FDA approved AUD medications
Fears about managing withdrawal symptoms	One page “cheat sheet” for identifying risk for severe withdrawal with recommendations to refer to substance use disorder specialty care if present
	Local clinical experts available for real-time consultation
	*All brief resources connected to more extensive follow-up materials that providers could access if interested
Inner setting: compatibility	
20 min appointments every 6 months allow insufficient time for diagnosis and monitoring	Worked with each site to identify procedures to connect with Primary Care/Mental Health Integration staff to assist with AUD diagnosis and providing regular follow-up
Characteristics of individuals: knowledge and beliefs about the intervention	
Lack of training and knowledge about substance use disorder in general and AUD diagnosis and pharmacotherapy specifically	Training provided through multiple educational sessions in large groups and small team meetings as well as available on the project web-site
Negative attitudes toward using medication to address substance use disorder	Frame pharmacotherapy as one option in treatment toolkit Provided multiple resources for multiple treatment options to allow patient choice
	Describe pharmacotherapy as possible “foot-in-the-door” for patients reluctant to engage in psychosocial treatments
Attitudes toward patients	
Generalize all AUD patients as unmotivated, highly complex, dishonest, etc.	Education provided on spectrum of AUD disorder and promote referral to specialty care for most severe and complex cases

### Aim 2: Identify variation in barriers across facilities in relation to variable implementation success

As seen in Table 1, the degree of change in prescribing rates differed across the three sites as did the significance of these changes. The analysis of AUD pharmacotherapy prescribing rate change following the intervention demonstrated that Site 1 had a non-significant trend toward increased prescribing, Site 2 had no change in prescribing rate and Site 3 had a significant increase in prescribing rate [31]. Given the differential intervention effects, we hypothesized that Site 2, which demonstrated no signal of an implementation intervention effect, would have additional identified barriers which would not be identified in Sites 1 and 3. Table 3 presents a joint display of site prescribing effects crossed with CFIR construct ratings.

As can be seen in Table 3, the only CFIR construct that fit the hypothesized pattern was Self-Efficacy. Self-Efficacy is defined as “individual’s belief in their own capabilities to execute courses of action to achieve implementation goals” [30]. While individual ratings from all three sites were mixed (as indicated by the *), a majority of respondents from Site 2 expressed serious reservations about their ability to competently prescribe medications for AUD.

“I mean, I would not be comfortable, I mean, I could address these saying that I can link you up with somebody that can talk about these and that these are some of the medications that can be used.” (Primary Care Provider, Site #2)

In contrast, the majority of respondents from Sites 1 and 3 expressed confidence in their ability to prescribing AUD medications, at least under certain conditions.

“Yeah, I actually had a patient that did the research and asked me about the medicine, so there you got someone who wants it, they are motivated, I think I definitely would. I think I would always try to get them into something else too, something in addition.” (Primary Care Provider, Site #3)

Interestingly, the more common pattern to emerge when examining the barriers by facility was that Site 2 and 3 had multiple common barriers that were not seen in Site 1. Constructs fitting this pattern included: (1) Relative Advantage (Innovation/Intervention Characteristics), (2) Design Quality and Packaging (Innovation/Intervention Characteristics), (3) Relative Priority (Inner Setting), and (4) Available Resources (Inner Setting).

Relative Advantage is defined as “stakeholders’ perception of the advantage of implementing the innovation versus an alternative solution” [30]. For Sites 2 and 3, most providers did not see an advantage to implementation of AUD pharmacotherapy in the primary care setting over referring all AUD patients to the specialty substance use disorders clinic in their facility. They felt that referrals to the specialty substance use disorders clinic were convenient for them and the patient, that patients who were truly ready for treatment would go to a separate clinic for treatment, and that the patients would benefit from the higher level of expertise and variety of interventions available in the specialty clinic. For example, this provider indicated a preference for referring patients to specialty care.

I feel like why change a system that’s not broken? I feel like we do have a really good Addiction Treatment Program here…other people [Addiction Treatment Program staff] do better than I do [treating substance use disorder] and it seems like the system is really effective. (Primary care provider, Site #2)

In contrast, providers from Site 1 recognized that stigma associated with seeking substance use disorder treatment and the additional steps of engaging with a separate clinic and a new provider may prevent some patients with AUD from following through with a specialty care referral. They viewed having some treatment services available in primary care as “meeting the patient where they are at” and “getting a foot in the door” for addressing AUD. This provider clarifies the impact of a separate program.

[With a] separate appointment at [Addiction Treatment Program], there’s going to be a big no-show rate getting them there versus having a primary care provider in the same appointment write that script. (Primary care provider, Site #1)

Design Quality and Packaging is defined as “perceived excellence in how the innovation is bundled, presented, and assembled” [30]. To clarify, comments coded here reflected on the materials developed as part of the implementation intervention. For Sites 2 and 3, while providers praised the content of the educational materials and the patient dashboard, they stated that they would not use the materials unless they were incorporated into the VHA computerized patient record system that the provider would already have open on their desktop during the patient visit. They stated that they would not open a separate website. (The logistics involved in adding on to the computerized patient record system did not make this option feasible during the timeframe of the intervention.)

I think unfortunately what you’ll probably find, there are a lot of websites we’re theoretically supposed to link out to, it just doesn’t work that well. If you could build whatever you want to tell people into the [computerized patient record system] you’re probably going to have much better luck. (Primary care provider, Site #3)

In contrast, providers at Site 1 simply did not bring up this concern. They also praised the materials and stated that they would be likely to use them during clinical encounters.

Well I would use the algorithms. And I would use the screening test. And I would use the initial template for treatment, for the medication, and I would use the follow-up too. (Primary care provider, Site #1)

Relative Priority is defined as “individuals’ shared perception of the importance of the implementation within the organization” [30]. For Sites 2 and 3, provider comments focused on other high priority issues within the VHA system that were demanding immediate attention and would limit the amount of time and energy available to focus on addressing AUD.

[AUD] probably ranks quite a bit below quite a lot of other things that are going on. Um, hepatitis C treatment, the opioid epidemic, access in general, those are probably the big ones. (Primary care provider, Site # 3)

Comments from providers in Site 1, on the other hand, tended to focus on the prioritization of addressing AUD coming from the highest levels of leadership in the facility:

The Chief Medical Officer has mentioned it, at least has mentioned alcohol, at every CMO meeting we’ve ever had with her. So I know that it’s a priority to her as well. (Primary care provider, Site #1)

Available Resources is defined as “the level of resources organizationally dedicated for implementation and on-going operations including physical space and time” [30]. Providers in Sites 2 and 3 focused on how they did not have the resources available in the primary care setting to follow-up with patients appropriately or to provide behavioral health support. They focused on primary care being overwhelmed in general, being “dumped on” from every direction, and not wanting to take responsibility for one more thing. This feeling of inadequate resources is reflected by this provider when asked about barriers to implementation:

Lack of support in terms of follow-up and monitoring side effects and assessing whether or not they’re continuing to use alcohol, support staff in terms of providing adequate follow up, time, it’s time consuming. (Primary care provider, Site #2)

While providers in Site 1 also focused on the importance of regular follow-up and behavioral health support, their comments tended to focus on the resources that were available to them that they could draw on to assist with implementation such as primary care mental health integration psychologists and tele-health care management programs:

I guess something to think about too is, you know, we have behavioral health embedded in primary care. It makes you wonder if we could use [embedded behavioral health providers] for the less complicated alcohol use disorders. If you could talk through the different medications, I’m going to give you a script for naltrexone, I’m also going to have you see the psychologist down the hall. I wonder if we could implement something like that. (Primary care provider, Site #1)

While it was expected that Site 2 would have identified barriers not found in Sites 1 and 3, this was only the case for Self-Efficacy suggesting that perhaps individual self-efficacy regarding implementation may be an important change ingredient. In fact, multiple barriers were identified in Site 2 and 3 that were not identified in Site 1. Both Site 2 and 3 providers did not identify an advantage to changing the way that patients with AUD were managed in their facility, expressed concern that the project materials were not integrated into the computerized patient record system that they used to manage their patients during clinical appointments, indicated that addressing AUD was not a high priority for their facility given competing high-profile priorities and indicated that they did not have the resources to support AUD treatment in the primary care setting.

Given that pre-implementation barriers were mostly not predictive of implementation success, another hypothesis regarding differential effectiveness of the intervention would be that process factors or differences in the way the actual implementation unfolded impacted implementation success. While it is beyond the scope of this paper to investigate process factors in-depth, Table 1 presents the total number of hours that the facility clinical champions reported dedicating to implementation on tracking sheets that they completed every week during the 12-month intervention period. This crude process measure does not demonstrate any relation to implementation success. Site 1 had the fewest identified barriers and the greatest number of dedicated champion hours, making their success not surprising. However, Site 3 which had several additional barriers and also had the lowest number of dedicated champion hours performed just as well. Future analysis of detailed champion facilitation meeting notes and post-implementation provider and champion interviews will further illuminate the role of process factors in implementation success.

## Discussion

The pre-implementation interviews served two purposes. First, they identified common barriers that informed refinements prior to the start of the implementation intervention and second, they were retroactively analyzed to identify variations in barriers that might assist in understanding the variable impact of the intervention across the three sites.

Four barriers that were common across sites were identified and refinements were made to the implementation intervention based on these identified barriers. While the impact of the intervention across sites was variable and not as substantial as hoped for, the research team felt the refinements made the intervention more palatable to primary care providers. Refinements were, however, limited by feasibility issues given the scope and timeframe of the project. For example, it was not possible to integrate the AUD dashboard into the computerized patient record system during the time frame of the project. Also, addressing broader, societal-level negative attitudes and generalizations about individuals with substance use disorders goes well beyond the project scope. Post-implementation interviews assessed primary care providers reactions to different aspects of the implementation intervention and analysis of this information will illuminate which aspects of the implementation intervention were most impactful and which barriers were left unaddressed. How this relates to the refinements made based on the pre-implementation interviews will add to the knowledge base regarding the value of extensive pre-implementation formative evaluations.

Examining variation in barriers, Self-Efficacy was the only construct that fit the expected pattern of being identified in Site 2, which did not increase prescribing rates, while not being present in Sites 1 and 3 which did increase prescribing rates. It is interesting to consider that perhaps the self-efficacy of the individuals whose behavior we are trying to influence may be more important to implementation success than the more concrete barriers related to aspects of the intervention itself or the setting in which the implementation is occurring. Based on this finding, in future implementation trials, we intend to conduct interviews assessing barriers to implementation and then ask respondents to rate, given the barriers they have just been discussing, the likelihood of implementation success.

It is also of interest that Site 2 and 3 had several additional barriers identified that were not present in Site 1 yet had very different implementation outcomes. At the present time, it is not clear why Site 3 was able to successfully improve their AUD pharmacotherapy prescribing rate although as mentioned above, Site 3 did have positive individual self-efficacy to implement changes, a facilitator they shared with Site 1 and that was not present in Site 2. In addition, the pre-implementation interviews, by definition, cannot provide information on CFIR Process variables. Data that remain to be analyzed include detailed champion facilitation meeting notes and post-implementation provider and local champion interviews. These data will allow us to assess differences in the implementation process across sites.

Currently, anecdotal information about the implementation process suggest that leadership turnover, resulting in loss of support for the project, as well as staff turnover, resulting in loss of institutional knowledge, may have interacted with pre-implementation barriers to drive the differential effects of the implementation intervention. At Site 2, primary care leadership turned over during the project and leadership support for the project following this turnover was diminished. The primary care mental health integration champion at this site was then assigned to multiple additional tasks, which did not allow her to focus on this project to the extent she desired. In addition, there was extensive provider turnover at Site 2 during the project so many providers who received educational offerings at the beginning of the project were no longer present resulting in a loss of institutional knowledge. Therefore, one important lesson learned from this project is that educational opportunities may be more effective if they are spread out and repeated across the life of the project rather than concentrated at the beginning. Finally, one of the site champions at Site 3 requested personal access to the provider panel dashboards and took dashboard output details to local clinical team meetings to alert providers personally to patients coming into their clinic in the next week, thereby circumventing their complaint that the dashboard was not available on the computerized patient record system that they had open during clinical appointments. This anecdotal information suggests that a strong implementation process may overcome initial barriers. Specifically, it suggests that supportive, consistent leadership and an enthusiastic, highly engaged champion may be able to overcome initial lack of provider support.

### Limitations

The major limitation to this study is its generalizability. First, facilities volunteered to participate and had a substance use disorder specialty care provider and a primary care mental health integration provider available and willing to serve in the role of champion for the project. Second, interview participants volunteered to participate based on an emailed request sent to all primary care providers. Therefore, these self-selected participants may have had stronger interest in or stronger opinions (positive or negative) regarding AUD treatment than would have been identified with a randomly selected sample. While this is true, the goal was to assess barriers likely to be encountered during implementation and the providers that were interviewed were not reluctant to share perceived barriers. Additionally, the interview guide asked providers about attitudes of their colleagues toward AUD treatment. A few shared that they were outliers among their colleagues in regards to their interest in seeing AUD treatment integrated into primary care settings and were able to comment on barriers their colleagues had communicated. Relatedly, demographic information was not collected from interview participants or non-participants so we are unable to assess if participants differed in systematic ways from those who did not participate. While there are clearly generalizability issues with this study, it is also clear that even under possibly idealized conditions, with champion resources readily available and interviewing providers that are potentially more positive toward the goals of the project, providers recognize multiple substantial barriers to integrating AUD pharmacotherapy into primary care settings. An addition limitation is that we are not at this time able to present detailed information regarding CFIR process constructs so the suggestion that differences in implementation outcomes are related to process variables is strictly conjecture at this time.

## Conclusions

This study supports the value and importance of understanding the pre-implementation context and illustrates how this understanding can be used to make refinements to the implementation intervention. It also highlights the importance of documenting and understanding the implementation process both to provide further data to understand differential effects as well as to examine whether implementation intervention refinements were well-received. The study adds to the literature supporting CFIR as a method to systematically identify and organize barriers to implementation in a way that can inform implementation intervention refinement and aid in the understanding of varying implementation results [33, 35]. Specifically, this study addressed two of the recommendations for advancing implementation science suggested by the CFIR developers in their recent systematic review: (1) CFIR was used throughout the research process in study design, data collection, and data analysis, and (2) CFIR was used to link determinants of implementation to outcomes [36]. Future steps will analyze data related to the implementation process for further clues to understanding the varying effect of the intervention.

The study’s quantitative and qualitative results highlight the incredible complexity involved in attempts to improve implementation of AUD pharmacotherapy. While some models have shown some success, the search continues for feasible, effective and replicable strategies to improve implementation. Extensive formative evaluation data adds to our arsenal of information regarding what will work where and for whom and adds to the knowledge base informing future efforts to implement pharmacological treatments for AUD as well as other evidence-based substance use disorder treatments that are currently vastly under-utilized.

## Additional files

**Additional file 1**. Semi-structured qualitative interview guide.

**Additional file 2**. Qualitative interview codebook.

## Data Availability

The datasets used and/or analyzed during the current study are available from the corresponding author on reasonable request.

## Abbreviations

AUD: alcohol use disorder
CFIR: Consolidated Framework for Implementation Research
VHA: Veterans Health Administration
