# Supplementary material for: Implementing alcohol use disorder pharmacotherapy in primary care settings: a qualitative analysis of provider-identified barriers and impact on implementation outcomes
Source: Addict Sci Clin Pract. 2019 Jul 10;14:24. doi: 10.1186/s13722-019-0151-7 (PMC6617941; doi:10.1186/s13722-019-0151-7)
Supplement: Supplementary file 1 — Additional file 1. Semi-structured qualitative interview guide. [file 13722_2019_151_MOESM1_ESM.docx]

**Primary Care Provider Pre-Implementation Interview**

[*Note send out materials/website ahead of time.]

**Now that we’ve finished the consenting process, we are ready to start the interview.**

**I am going to turn on the digital recorder now. [Turn on audio.]**

**[Say into digital recorder:]**

**This is participant number __________.**

**The date is _____________.**

**This is interviewer (your first name).**

**I am interviewing you today because we are beginning a project to provide education and support to primary care providers in addressing alcohol use disorders (AUDs) with their patients. We are interested in finding out how you currently address AUDs in your practice and getting your opinions about educational materials and support tools that we are planning to use in our project.**

**To start with, I am going to tell you about a case and I would like you to walk me through how you would approach this.**

1. A middle-aged man shows up for routine follow up of diabetes. He has screened positive for risky alcohol use. What do you do?
   1. Do you address drinking goals with the patient? (Note: you want to get at whether they engage the patient in setting the goal and whether they would except a reduction goal)
      1. (If the provider only mentions abstinence as an appropriate goal) If the patient is not willing to consider abstinence but is willing to consider reducing drinking, are you willing to work with them toward that goal?

2. **(If provider states they would gather more information, do a more thorough assessment.)** Assuming that you identify that the patient needs services for alcohol use disorder, what treatment suggestions might you make?

a. How would you talk with your patient about your suggestions?

3. **(If provider states that they would refer the patient to another clinic.)** Whom would you contact?

a. How would you interact with the provider/clinic that you contacted in coordinating care for the patient?

b. How would you document referral in your notes?

c. Do you follow up to ensure the patient followed through with treatment recommendations? If yes, how?

**Next, I’d like to find out your opinions on the provider support materials that we are developing for the project. Did you receive the materials we sent in advance? Did you get a chance to review them?**

1. **I’d like to walk you through these materials (that I sent you) and then I will ask you some questions about them. [Walk participant through materials.]**
   1. What in these materials looks the most helpful to you?
   2. Anything you don’t like?
   3. Is there anything missing that you would like to see added to the materials?
   4. Do you see yourself using these materials? If so, what would you use?
   5. Are there pieces of this information that would be helpful to have in hard copy?
   6. In your opinion, what would be the most effective way to introduce these materials to providers? E.G., educational presentation at Grand Rounds, small group discussion during team meeting, review on your own
2. Prior to receiving the materials, were you aware that there are medications that help people reach their goals to reduce or stop drinking? (Knowledge & Beliefs about the Intervention)
   1. (If no, skip to Question 4) In your opinion, how strong is the research evidence supporting the effectiveness of these medications? (Evidence Strength & Quality)
3. Have you ever prescribed one of these AUD medications? (Individual Stage of Change)
   1. **If yes, ask follow-up questions:**
      1. How did you feel it worked? (Knowledge & Beliefs about the Intervention)
      2. What additional support or information would you have liked to have? (Access to Knowledge & Information)
   2. **If no, ask follow up questions:**
      1. What are some reasons you don’t prescribe AUD medications?
      2. What would you need to know about AUD medications use in order to feel comfortable prescribing one? (Access to Knowledge & Information)
      3. What supports would you like to have in place if you were going to prescribe an AUD medication?
      4. Do you think you would be willing to try it out with a few patients to see how it works? (Trialability)
4. How confident are you in your ability to integrate alcohol use disorder medications into your practice? (Self-Efficacy)

**These next few questions refer to other primary care providers in your clinic.**

1. How comfortable do you think your primary care colleagues are working with patients who have alcohol use disorders?
2. Are you aware of any other primary care providers in your facility that are prescribing medications for alcohol use disorders?
   1. If yes, how many?
3. How do you think other primary care providers in your facility would feel about prescribing alcohol use disorder medications? (Compatibility)
4. What major barriers do you think will be encountered in trying to encourage primary care providers to prescribe medications for alcohol use disorders? (Complexity)
5. Thinking about how patients with alcohol use disorders are currently managed, do you see advantages to encouraging primary care providers to prescribe medication for alcohol? What about disadvantages?

**We also plan to mail educational materials to patients. Here is the educational brochure that we plan to send out. Please, take a few minutes to look it over and then I will ask you some questions about the brochure. (Note: This will be the first time they see these materials.)**

1. What concerns, if any, do you have about patients receiving these materials in the mail?
2. Is there any information missing that you would like to see added to the brochure?
3. We also plan to send this introduction letter with the brochure. What do you think about this cover letter? (If needed, prompt: Doe sit address concerns mentioned above?)
4. Do you think any information should be added to the cover letter?
5. Do you have any suggestions about other ways to get this information out to patients?

**We will also be emailing providers to let them know when the mailing will go out. This will be sometime in March or April.**

**I have just a few questions regarding any other efforts to address AUDs in primary care.**

1. Outside of this project, are you aware of any national or local VA efforts to address AUDs in primary care or to promote prescribing of AUD medications? If yes, please tell me a little bit about those. (External Policy & Incentives, Tension for Change)
2. Do you believe there is any pressure on your local facility to either catch up or move ahead of other facilities in addressing AUDs in primary care or in implementing AUD pharmacotherapy? (Peer Pressure, Tension for Change)
3. Overall, how high of a priority do you think addressing AUD in primary care setting is for your facility at this time? (Relative Priority)
   1. What other quality improvement initiatives are in progress now?
   2. How might these affect the ability to implement alcohol use disorder pharmacotherapy now?
4. Are you aware of any incentives for addressing AUD in primary care settings, for example, would it be considered during performance reviews or associated with a performance award? (Organizational Incentives & Rewards)

**Thank you for taking the time to speak with me today.**

1. Is there anything I didn’t specifically ask about that would be helpful for me to know?
2. Do you have any questions for me?
