# Supplementary material for: Implementing alcohol use disorder pharmacotherapy in primary care settings: a qualitative analysis of provider-identified barriers and impact on implementation outcomes
Source: Addict Sci Clin Pract. 2019 Jul 10;14:24. doi: 10.1186/s13722-019-0151-7 (PMC6617941; doi:10.1186/s13722-019-0151-7)
Supplement: Supplementary file 2 — Additional file 2. Qualitative interview codebook. [file 13722_2019_151_MOESM2_ESM.docx]

**ADAPT-PC CFIR Codebook**

- **Additions to original CFIR codebook noted in bold text**

| 1. **Innovation Characteristics** |  |
| --- | --- |
| 1. Innovation Source | Definition: Perception of key stakeholders about whether the innovation is externally or internally developed.  Inclusion Criteria: Include statements about the source of the innovation and the extent to which interviewees view the change as internal to the organization, e.g., an internally developed program, or external to the organization, e.g., a program coming from the outside. Note: May code and rate as "I" for internal or "E" for external.  Exclusion Criteria: Exclude or double code statements related to who participated in the decision process to implement the innovation to [Engaging](http://cfirwiki.net/wiki/index.php?title=Engaging), as an indication of early (or late) engagement. Participation in decision-making is an effective engagement strategy to help people feel ownership of the innovation. |
| 1. Evidence Strength & Quality | Definition: Stakeholders’ perceptions of the quality and validity of evidence supporting the belief that the innovation will have desired outcomes.  Inclusion Criteria: Include statements regarding awareness of evidence and the strength and quality of evidence, as well as the absence of evidence or a desire for different types of evidence, such as pilot results instead of evidence from the literature.   - **Comments on existing evidence**   Exclusion Criteria: Exclude or double code statements regarding the receipt of evidence as an engagement strategy to [Engaging](http://cfirwiki.net/wiki/index.php?title=Engaging): Key Stakeholders.  Exclude or double code descriptions of use of results from local or regional pilots to [Trialability](http://cfirwiki.net/wiki/index.php?title=Trialability). |
| 1. Relative Advantage | Definition: Stakeholders’ perception of the advantage of implementing the innovation versus an alternative solution.  Inclusion Criteria: Include statements that demonstrate the innovation is better (or worse) than existing programs.  Exclusion Criteria: Exclude statements that demonstrate a strong need for the innovation and/or that the current situation is untenable and code to [Tension for Change](http://cfirwiki.net/wiki/index.php?title=Tension_for_Change). |
| 1. Adaptability | Definition: The degree to which an innovation can be adapted, tailored, refined, or reinvented to meet local needs.  Inclusion Criteria: Include statements regarding the (in)ability to adapt the innovation to their context, e.g., complaints about the rigidity of the protocol. Suggestions for improvement can be captured in this code but should not be included in the rating process, unless it is clear that the participant feels the change is needed but that the program cannot be adapted. However, it may be possible to infer that a large number of suggestions for improvement demonstrates lack of compatibility, see exclusion criteria below.  Exclusion Criteria: Exclude or double code statements that the innovation did or did not need to be adapted to [Compatibility](http://cfirwiki.net/wiki/index.php?title=Compatibility). |
| 1. Trialability | Definition: The ability to test the innovation on a small scale in the organization, and to be able to reverse course (undo implementation) if warranted.  Inclusion Criteria: Include statements related to whether the site piloted the innovation in the past or has plans to in the future, and comments about whether they believe it is (im)possible to conduct a pilot.  Exclusion Criteria: Exclude or double code descriptions of use of results from local or regional pilots to [Evidence Strength & Quality](http://cfirwiki.net/wiki/index.php?title=Evidence_Strength_%26_Quality). |
| 1. Complexity | Definition: Perceived difficulty of the innovation, reflected by duration, scope, radicalness, disruptiveness, centrality, and intricacy and number of steps required to implement.   - **Within intervention (steps, materials, etc)**   Inclusion Criteria: Code statements regarding the complexity of the innovation itself.  Exclusion Criteria: Exclude statements regarding the complexity of implementation and code to the appropriate CFIR code, e.g., difficulties related to space are coded to Available Resources and difficulties related to engaging participants in a new program are coded to [Engaging](http://cfirwiki.net/wiki/index.php?title=Engaging): Innovation Participants. |
| 1. Design Quality & Packaging | Definition: Perceived excellence in how the innovation is bundled, presented, and assembled.  Inclusion Criteria: Include statements regarding the quality of the materials and packaging.   - **Including training** - **Including training they want** - **Include other patient resources**   Exclusion Criteria: Exclude statements regarding the presence or absence of materials and code to [Available Resources](http://cfirwiki.net/wiki/index.php?title=Available_Resources).  Exclude statements regarding the receipt of materials as an engagement strategy and code to [Engaging](http://cfirwiki.net/wiki/index.php?title=Engaging). |
| 1. Cost | Definition: Costs of the innovation and costs associated with implementing the innovation including investment, supply, and opportunity costs.  Inclusion Criteria: Include statements related to the cost of the innovation and its implementation.  Exclusion Criteria: Exclude statements related to physical space and time, and code to [Available Resources](http://cfirwiki.net/wiki/index.php?title=Available_Resources). In a research study, exclude statements related to costs of conducting the research components (e.g., funding for research staff, participant incentives). |
| 1. **Outer Setting** |  |
| 1. Needs & Resources of Those Served by the Organization | Definition: The extent to which the needs of those served by the organization (e.g., patients), as well as barriers and facilitators to meet those needs, are accurately known and prioritized by the organization.  Inclusion Criteria: Include statements demonstrating (lack of) awareness of the needs and resources of those served by the organization. Analysts may be able to infer the level of awareness based on statements about: 1. Perceived need for the innovation based on the needs of those served by the organization and if the innovation will meet those needs; 2. Barriers and facilitators of those served by the organization to participating in the innovation; 3. Participant feedback on the innovation, i.e., satisfaction and success in a program. In addition, include statements that capture whether or not awareness of the needs and resources of those served by the organization influenced the implementation or adaptation of the innovation.  Exclusion Criteria: Exclude statements that demonstrate a strong need for the innovation and/or that the current situation is untenable and code to [Tension for Change](http://cfirwiki.net/wiki/index.php?title=Tension_for_Change).  Exclude statements related to engagement strategies and outcomes, e.g., how innovation participants became engaged with the innovation, and code to [Engaging](http://cfirwiki.net/wiki/index.php?title=Engaging): Innovation Participants. |
| 1. Cosmopolitanism | Definition: The degree to which an organization is networked with other external organizations.  Inclusion Criteria: Include descriptions of outside group memberships and networking done outside the organization.  Exclusion Criteria: Exclude statements about general networking, communication, and relationships in the organization, such as descriptions of meetings, email groups, or other methods of keeping people connected and informed, and statements related to team formation, quality, and functioning, and code to [Networks & Communications](http://cfirwiki.net/wiki/index.php?title=Networks_%26_Communications). |
| 1. Peer Pressure | Definition: Mimetic or competitive pressure to implement an innovation, typically because most or other key peer or competing organizations have already implemented or are in a bid for a competitive edge.  Inclusion Criteria: Include statements about perceived pressure or motivation from other entities or organizations in the local geographic area or system to implement the innovation.  Exclusion Criteria: |
| 1. External Policy & Incentives | Definition: A broad construct that includes external strategies to spread innovations including policy and regulations (governmental or other central entity), external mandates, recommendations and guidelines, pay-for-performance, collaboratives, and public or benchmark reporting.  Inclusion Criteria: Include descriptions of external performance measures from the system.  Exclusion Criteria: |
| 1. **Inner Setting** |  |
| 1. Structural Characteristics | Definition: The social architecture, age, maturity, and size of an organization.  Inclusion Criteria:  Exclusion Criteria: |
| 1. Networks & Communications | Definition: The nature and quality of webs of social networks, and the nature and quality of formal and informal communications within an organization.  Inclusion Criteria: Include statements about general networking, communication, and relationships in the organization, such as descriptions of meetings, email groups, or other methods of keeping people connected and informed, and statements related to team formation, quality, and functioning.  Exclusion Criteria: Exclude statements related to implementation leaders' and users' access to knowledge and information regarding using the program, i.e., training on the mechanics of the program and code to [Access to Knowledge & Information](http://cfirwiki.net/wiki/index.php?title=Access_to_Knowledge_%26_Information).  Exclude statements related to engagement strategies and outcomes, e.g., how key stakeholders became engaged with the innovation and what their role is in implementation, and code to [Engaging](http://cfirwiki.net/wiki/index.php?title=Engaging): Key Stakeholders.  Exclude descriptions of outside group memberships and networking done outside the organization and code to [Cosmopolitanism](http://cfirwiki.net/wiki/index.php?title=Cosmopolitanism). |
| 1. Culture | Definition: Norms, values, and basic assumptions of a given organization.  Inclusion Criteria: Inclusion criteria, and potential sub-codes, will depend on the framework or definition used for “culture.” For example, if using the [Competing Values Framework](http://www.implementationscience.com/content/2/1/13/abstract) (CVF), you may include four sub-codes related to the four dimensions of the CVF and code statements regarding one or more of the four dimension in an organization.  Exclusion Criteria: |
| 1. Implementation Climate | Definition: The absorptive capacity for change, shared receptivity of involved individuals to an innovation, and the extent to which use of that innovation will be rewarded, supported, and expected within their organization.  Inclusion Criteria: Include statements regarding the general level of receptivity to implementing the innovation.  Exclusion Criteria: Exclude statements regarding the general level of receptivity that are captured in the sub-codes. |
| 1. Tension for Change | Definition: The degree to which stakeholders perceive the current situation as intolerable or needing change.  Inclusion Criteria: Include statements that (do not) demonstrate a strong need for the innovation and/or that the current situation is untenable, e.g., statements that the innovation is absolutely necessary or that the innovation is redundant with other programs. Note: If a participant states that the innovation is redundant with a preferred existing program, (double) code lack of [Relative Advantage](http://cfirwiki.net/wiki/index.php?title=Relative_Advantage), see exclusion criteria below.  Exclusion Criteria: Exclude statements regarding specific needs of individuals that demonstrate a need for the innovation, but do not necessarily represent a strong need or an untenable status quo, and code to [Needs and Resources of Those Served by the Organization.](http://cfirwiki.net/wiki/index.php?title=Patient_Needs_%26_Resources)  Exclude statements that demonstrate the innovation is better (or worse) than existing programs and code to [Relative Advantage](http://cfirwiki.net/wiki/index.php?title=Relative_Advantage). |
| 1. Compatibility | Definition: The degree of tangible fit between meaning and values attached to the innovation by involved individuals, how those align with individuals’ own norms, values, and perceived risks and needs, and how the innovation fits with existing workflows and systems.   - **Only code when participate makes the connection with the intervention, e.g., prescribing AUD medication in PC setting, not generally talking about AUD with patients.**   Inclusion Criteria: Include statements that demonstrate the level of compatibility the innovation has with organizational values and work processes. Include statements that the innovation did or did not need to be adapted as evidence of compatibility or lack of compatibility.  Exclusion Criteria: Exclude or double code statements regarding the priority of the innovation based on compatibility with organizational values to [Relative Priority](http://cfirwiki.net/wiki/index.php?title=Relative_Priority), e.g., if an innovation is not prioritized because it is not compatible with organizational values. |
|  |  |
| 1. Relative Priority | Definition: Individuals’ shared perception of the importance of the implementation within the organization.   - **PC addressing alcohol. Can include comments regarding relative priority for patients they serve as well as comments regarding relative priority for the organization. These may be conflicting.**   Inclusion Criteria: Include statements that reflect the relative priority of the innovation, e.g., statements related to change fatigue in the organization due to implementation of many other programs.  Exclusion Criteria: Exclude or double code statements regarding the priority of the innovation based on compatibility with organizational values to [Compatibility](http://cfirwiki.net/wiki/index.php?title=Compatibility), e.g., if an innovation is not prioritized because it is not compatible with organizational values. |
| 1. Organizational Incentives & Rewards | Definition: Extrinsic incentives such as goal-sharing, awards, performance reviews, promotions, and raises in salary, and less tangible incentives such as increased stature or respect.  Inclusion Criteria: Include statements related to whether organizational incentive systems are in place to foster (or hinder) implementation, e.g., rewards or disincentives for staff engaging in the innovation.  Exclusion Criteria: |
| 1. Goals & Feedback | Definition: The degree to which goals are clearly communicated, acted upon, and fed back to staff, and alignment of that feedback with goals.  Inclusion Criteria: Include statements related to the (lack of) alignment of implementation and innovation goals with larger organizational goals, as well as feedback to staff regarding those goals, e.g., regular audit and feedback showing any gaps between the current organizational status and the goal. Goals and Feedback include organizational processes and supporting structures independent of the implementation process. Evidence of the integration of evaluation components used as part of “Reflecting and Evaluating” into on-going or sustained organizational structures and processes may be (double) coded to Goals and Feedback.  Exclusion Criteria: Exclude statements that refer to the implementation team’s (lack of) assessment of the progress toward and impact of implementation, as well as the interpretation of outcomes related to implementation, and code to [Reflecting & Evaluating](http://cfirwiki.net/wiki/index.php?title=Reflecting_%26_Evaluating). Reflecting and Evaluating is part of the implementation process; it likely ends when implementation activities end. It does not require goals be explicitly articulated; it can focus on descriptions of the current state with real-time judgment, though there may be an implied goal (e.g., we need to implement the innovation) when the implementation team discusses feedback in terms of adjustments needed to complete implementation. |
| 1. Learning Climate | Definition: A climate in which: 1. Leaders express their own fallibility and need for team members’ assistance and input; 2. Team members feel that they are essential, valued, and knowledgeable partners in the change process; 3. Individuals feel psychologically safe to try new methods; and 4. There is sufficient time and space for reflective thinking and evaluation.  Inclusion Criteria: Include statements that support (or refute) the degree to which key components of an organization exhibit a “learning climate.”  Exclusion Criteria: |
| 1. Readiness for Implementation | Definition: Tangible and immediate indicators of organizational commitment to its decision to implement an innovation.  Inclusion Criteria: Include statements regarding the general level of readiness for implementation.  Exclusion Criteria: Exclude statements regarding the general level of readiness for implementation that are captured in the sub-codes. |
| 1. Leadership Engagement | Definition: Commitment, involvement, and accountability of leaders and managers with the implementation of the innovation.  Inclusion Criteria: Include statements regarding the level of engagement of organizational leadership.  Exclusion Criteria: Exclude or double code statements regarding leadership engagement to Engaging: [Formally Appointed Internal Implementation Leaders](http://cfirwiki.net/wiki/index.php?title=Formally_Appointed_Internal_Implementation_Leaders) or [Champions](http://cfirwiki.net/wiki/index.php?title=Champions) *if* an organizational leader is also an implementation leader, e.g., if a director of primary care takes the lead in implementing a new treatment guideline. Note that a key characteristic of this Implementation Leader/Champion is that s/he is also an Organizational Leader. |
| 1. Available Resources | Definition: The level of resources organizational dedicated for implementation and on-going operations including physical space and time.   - **Give us what we need to do it** - **Staff, MH resources, etc** - **Do providers have what they need to implement** - **They have to connect this to the intervention, e.g., whether or not time or staff would be available for THIS, not general comments about “we don’t have enough time”, “we could use more help from mental health”.**   Inclusion Criteria: Include statements related to the presence or absence of resources specific to the innovation that is being implemented.  Exclusion Criteria: Exclude statements related to training and education and code to [Access to Knowledge & Information](http://cfirwiki.net/wiki/index.php?title=Access_to_Knowledge_%26_Information).  Exclude statements related to the quality of materials and code to [Design Quality & Packaging](http://cfirwiki.net/wiki/index.php?title=Design_Quality_%26_Packaging).  In a research study, exclude statements related to resources needed for conducting the research components (e.g., time to complete research tasks, such as IRB applications, consenting patients). |
| 1. Access to Knowledge & Information | Definition: Ease of access to digestible information and knowledge about the innovation and how to incorporate it into work tasks.  Inclusion Criteria: Include statements related to implementation leaders' and users' access to knowledge and information regarding use of the program, i.e., training on the mechanics of the program.  Exclusion Criteria: Exclude statements related to engagement strategies and outcomes, e.g., how key stakeholders became engaged with the innovation and what their role is in implementation, and code to [Engaging](http://cfirwiki.net/wiki/index.php?title=Engaging): Key Stakeholders.  Exclude statements about general networking, communication, and relationships in the organization, such as descriptions of meetings, email groups, or other methods of keeping people connected and informed, and statements related to team formation, quality, and functioning, and code to [Networks & Communications](http://cfirwiki.net/wiki/index.php?title=Networks_%26_Communications). |
| 1. **Characteristics of Individuals** |  |
| 1. Knowledge & Beliefs about the Innovation | Definition: Individuals’ attitudes toward and value placed on the innovation, as well as familiarity with facts, truths, and principles related to the innovation.   - **Comments on their own personal knowledge of the intervention, e.g., they may view the “strength of evidence” as high but admit limited personal knowledge** - **Related to medication** - **The handling of AUD in PC**   Inclusion Criteria:  Exclusion Criteria: Exclude statements related to familiarity with evidence about the innovation and code to [Evidence Strength & Quality](http://cfirwiki.net/wiki/index.php?title=Evidence_Strength_%26_Quality). |
| 1. Self-efficacy | Definition: Individual belief in their own capabilities to execute courses of action to achieve implementation goals.  Inclusion Criteria:  Exclusion Criteria: |
| 1. Individual Stage of Change | Definition: Characterization of the phase an individual is in, as s/he progresses toward skilled, enthusiastic, and sustained use of the innovation.  Inclusion Criteria:  Exclusion Criteria: |
| 1. Individual Identification with Organization | Definition: A broad construct related to how individuals perceive the organization, and their relationship and degree of commitment with that organization.  Inclusion Criteria:  Exclusion Criteria: |
| 1. Other Personal Attributes | Definition: A broad construct to include other personal traits such as tolerance of ambiguity, intellectual ability, motivation, values, competence, capacity, and learning style.   - **Providers attitudes towards patients, e.g., generalizing comments about patients with AUD such as “they will lie”, “they are not motivated”** - **Providers approach (authoritarian vs patient centered)**   Inclusion Criteria:  Exclusion Criteria: |

**General Coding Rules:**

When two codes are in question for a passage, consider the primary meaning of the passage to assign code; consider what the participant is truly saying. Analysts may wish to err on the side of inclusion or double coding.

**General Rating Rules:**

| **Ratings** | | | | | | |
| --- | --- | --- | --- | --- | --- | --- |
| M | -2 | -1 | 0 | X | +1 | +2 |

In general, ratings are determined based on two factors: 1) valence and 2) strength.

**Valence: positive or negative influence on implementation**

*Rating component: X, 0, +, -*

The valence component of a rating is determined by the influence the coded data has on the implementation process, i.e., contextual factors that facilitate or hinder implementation. Due to limited data, analysts may have to infer the influence on implementation based on simple presence or absence of a construct. For example, if a participant states that the intervention has advantages over existing programs, but does not state how this has influenced implementation, the analyst can infer that the presence of relative advantage facilitated implementation. However, whenever the data allows, the analysts should apply ratings based on the influence the construct has on implementation, not the presence or absence of a construct; presence or absence of a positive construct (e.g. relative advantage) does not always constitute a matching positive or negative influence on implementation.

In the event that comments are mixed, i.e., some comments are negative and some comments are positive, try to tip the rating to a weak positive or weak negative, based on the aggregate of the comments. However, if you feel the comments are equally positive and negative, apply a mixed (X) rating. Some users of the CFIR have denoted level of agreement among participants in their rating by adding a * to the rating if comments were mixed. For example, if the aggregate of mixed comments was positive, the rating was +1*. Some users feel it’s important to record discord among participants because it indicates a negative influence on implementation.

In the event that the comments are neutral, i.e., comments are related to a construct but have no bearing on the implementation, apply the neutral (0) rating.

**Strength: weak or strong influence on implementation**

*Rating component: 1, 2*

The strength component of a rating is determined by a number of factors, including: level of agreement among participants, strength of language, and use of concrete examples. However, sometimes analysts may choose to apply relative ratings, versus absolute ratings, in order to differentiate between organization in the study.
